# Current Status of Latency Reversing Agents Facing the Heterogeneity of HIV-1 Cellular and Tissue Reservoirs

**DOI:** 10.3389/fmicb.2019.03060

**Published:** 2020-01-24

**Authors:** Amina Ait-Ammar, Anna Kula, Gilles Darcis, Roxane Verdikt, Stephane De Wit, Virginie Gautier, Patrick W. G. Mallon, Alessandro Marcello, Olivier Rohr, Carine Van Lint

**Affiliations:** ^1^Service of Molecular Virology, Department of Molecular Virology (DBM), Université Libre de Bruxelles (ULB), Gosselies, Belgium; ^2^Malopolska Centre of Biotechnology, Laboratory of Virology, Jagiellonian University, Krakow, Poland; ^3^Infectious Diseases Department, Liège University Hospital, Liège, Belgium; ^4^Service des Maladies Infectieuses, CHU Saint-Pierre, Université Libre de Bruxelles, Bruxelles, Belgium; ^5^UCD Centre for Experimental Pathogen Host Research (CEPHR), School of Medicine, University College Dublin, Dublin, Ireland; ^6^Laboratory of Molecular Virology, International Centre for Genetic Engineering and Biotechnology (ICGEB), Trieste, Italy; ^7^Université de Strasbourg, EA7292, FMTS, IUT Louis Pasteur, Schiltigheim, France

**Keywords:** HIV-1, latency reversing agents, cure, latency, reservoirs, heterogeneity

## Abstract

One of the most explored therapeutic approaches aimed at eradicating HIV-1 reservoirs is the “shock and kill” strategy which is based on HIV-1 reactivation in latently-infected cells (“shock” phase) while maintaining antiretroviral therapy (ART) in order to prevent spreading of the infection by the neosynthesized virus. This kind of strategy allows for the “kill” phase, during which latently-infected cells die from viral cytopathic effects or from host cytolytic effector mechanisms following viral reactivation. Several latency reversing agents (LRAs) with distinct mechanistic classes have been characterized to reactivate HIV-1 viral gene expression. Some LRAs have been tested in terms of their potential to purge latent HIV-1 *in vivo* in clinical trials, showing that reversing HIV-1 latency is possible. However, LRAs alone have failed to reduce the size of the viral reservoirs. Together with the inability of the immune system to clear the LRA-activated reservoirs and the lack of specificity of these LRAs, the heterogeneity of the reservoirs largely contributes to the limited success of clinical trials using LRAs. Indeed, HIV-1 latency is established in numerous cell types that are characterized by distinct phenotypes and metabolic properties, and these are influenced by patient history. Hence, the silencing mechanisms of HIV-1 gene expression in these cellular and tissue reservoirs need to be better understood to rationally improve this cure strategy and hopefully reach clinical success.

## Introduction

Approximately, 36.9 million people in the world are living with HIV, while there were 33.3 million in 2010, showing that HIV is still a global public health problem (UNAIDS 2018).

The development of ART has allowed the suppression of virus replication to undetectable levels. Thus, ART reduces HIV-associated morbidity, prolongs survival, prevents transmission, and results in a partial reconstitution of the immune system, as measured by an increase in circulating CD4^+^ T cells. However, ART can cause cumulative toxic effects with the emergence of HIV-1 drug-resistant variants (Hu and Kuritzkes, 2014). Moreover, ART is unable to achieve complete virus eradication since it targets only actively replicating virus, and as such, treatment needs to be taken life-long. The main barrier to an HIV cure is the formation of stable reservoirs of latent HIV-1 that are defined as a cell type or an anatomical site in which integrated proviruses persist (Eisele and Siliciano, 2012). The latent reservoirs are highly heterogeneous and composed of multiple cell types, such as macrophages, but the best-characterized ones are a small population of HIV-1-infected memory CD4^+^ T cells. In addition to cellular reservoirs, GALT, CNS, genital tract, and lymph nodes are the main anatomical reservoirs of HIV-1 (Coombs et al., 2003; Gras and Kaul, 2010; Yukl et al., 2013). The role of the respiratory tract (Cribbs et al., 2015), liver (Penton and Blackard, 2014), kidney (Winston et al., 2001), and adipose tissue (Couturier et al., 2015) as HIV-1 reservoirs is also gaining importance. Moreover, an anatomical site in which ART penetration is limited (called a sanctuary) or a site which is immune-privileged (such as B cell follicular centers within lymph nodes, testis, and the brain) may allow for residual replication contributing to viral persistence (Connick et al., 2007; Chomont et al., 2009; Buzón et al., 2011; Eisele and Siliciano, 2012; Fletcher et al., 2014). Together, these reservoirs can be induced to actively produce viruses by various cellular stimuli and therefore represent one potential source of rebound of viremia after ART interruption (Chun et al., 1997; Davey et al., 1999; Beliakova-Bethell et al., 2017; Ganor et al., 2019).

Viruses have developed different mechanisms to escape the host immune system. In addition to the rapid evolution of viral variants, the establishment of viral latency in the infected cells is one of these mechanisms (Deng et al., 2015). Latently-infected cells contain stably-integrated, replication-competent proviruses repressed by a plethora of silencing mechanisms operating at the transcriptional and post-transcriptional levels. The number of latently-infected cells carrying replication-competent proviruses is extremely low with a majority of defective proviruses (Ho et al., 2013; Bruner et al., 2016, 2019). Significant progress has been made in the development of various therapeutic approaches that target HIV-1 and prevent disease progression. Currently, two strategies are under investigation in order to reach long-term control of viral replication in the absence of ART: the first strategy is aiming at achieving a sterilizing cure (i.e., a total elimination of the virus from the human body), whereas the second is a functional cure (i.e., a remission or long-term control of HIV-1 in the absence of ART, without loss of CD4^+^ T cells, no clinical progression, lack of HIV-1 transmission, and a reduction of the size of the reservoirs). Given the difficulty of achieving a sterilizing cure, one specific approach for a functional cure, called the “shock and kill” strategy, has become one of the major focus of attention (Thorlund et al., 2017). However, it now appears that the main barrier to reaching success with this “shock and kill” strategy is the heterogeneity of the latent HIV-1 reservoirs that is reflected by the diversity of infected cell types residing in the blood and in the different tissues and by the complexity of the molecular mechanisms governing latency and most likely differing from one cell to the other.

The present review will discuss the heterogeneity of the HIV-1 reservoirs that has been highlighted by studies using LRAs. We will describe the determinants responsible for these heterogeneous responses to LRAs, including the diversity of cell types composing the reservoirs (their origin, their differentiation, and their activation state). We will briefly present the multiple molecular mechanisms governing latency, the HIV-1 diversity within the latent and reactivated reservoirs, the patient-to-patient and cellular variations in response to LRAs. Finally, we will discuss why a better understanding of these elements is crucial for reaching an HIV-cure.

## Heterogeneous Composition of HIV-1 Reservoirs

### CD4^+^ T Cell Reservoirs

HIV-1 infects subsets of CD4^+^ T lymphocytes, leading either to productively-infected cells or, rarely, to latently-infected cells. HIV-1 infected CD4^+^ T cells can be distinguished either by their state of differentiation, by their function, or by the markers they express on their surface.

#### CD4^+^ T Cell Differentiation Subsets

CD4^+^ T cells infected with HIV-1 can be grouped by their state of differentiation into naive T cells (T_N_, CD45RA^+^, CD62L^+^, and CD95^–^) and memory CD4^+^ T cells, which can be further divided into four subpopulations: central memory T cells (T_CM_, CD45RA^–^, CCR7^+^, and CD27^+^), effector memory T cells (T_EM_, CD45RA^–^, CCR7^–^, and CD27^–^), transitional memory T cells (T_TM_, CD45RA^–^, CCR7^–^, and CD27^+^), and stem cell-like memory T cells (T_SCM_, CD45RA^+^, CCR7^+^, CD27^+^, and CD95^+^). T_N_ cells that are more resistant to HIV-1 infection than memory CD4^+^ T cells and as such contain lower levels of HIV-1 DNA (Venanzi Rullo et al., 2019), produce as many virions (Zerbato et al., 2019) or greater (Venanzi Rullo et al., 2019) amount of replication-competent HIV-1 than the memory CD4^+^ T cells following latency reversal with LRAs, indicating that T_N_ cells are an important reservoir of latent HIV-1. Moreover, the total proportion of T_N_ increases under ART regimen compared to memory T cells (Wightman et al., 2010). However, clonal expansion is more frequently observed in memory than in T_N_ cells, thus contributing to HIV-1 persistence (von Stockenstrom et al., 2015). Indeed, homeostatic proliferation driving clonal expansion has been evidenced by pioneering work of Chomont et al. (2009) that identified T_CM_ and T_TM_ as the main reservoir for HIV-1 infection. These two major subgroups are a more stable reservoir for HIV-1 than T_EM_ which are short-lived and, unlike T_CM_ and T_TM_, express markers of activation (HLA-DR) (Pardons et al., 2019) and might be more sensitive to programmed cell death (Riou et al., 2007; Chomont et al., 2009; Cockerham et al., 2014). It has been previously shown by the group of Sarah Palmer that T_EM_ which are in an activation state carry the highest number of intact proviruses compared to T_CM_, T_TM_, and T_N_ (Hiener et al., 2017). This finding suggests that the resting phenotype of CD4^+^ T cells (i.e., characterized by no expression of activation markers) is not a prerequisite for latent infection. These differences could be explained by patient-specific variations related to the duration of infection before ART initiation or to the duration of ART. Moreover, some evidence supports the theory that HIV-1 can establish latent infection in actively replicating CD4^+^ T cells, suggesting that HIV-1 infection of both resting and activated primary CD4^+^ T cells could result in latency (Chavez et al., 2015). The T_TD_, corresponding to aged T-cell populations that reflect HIV-1 disease progression (Cao et al., 2009) and in which integrated HIV-1 DNA is also detected, are a very small reservoir with a reduced frequency in HIV-1 individuals under suppressive ART and improved CD4^+^ T cell counts (Chomont et al., 2009; Behrens et al., 2018). Finally, T_SCM_ are permissive to HIV-1 infection and contribute to its persistence by their capacity of self-renewal and prolonged survival rate. The proportion of viral DNA associated with these T_SCM_ cells is higher than in T_CM_. Although T_SCM_ are latently-infected, they represent only a small fraction of the total reservoir (Gattinoni et al., 2011; Buzon et al., 2014).

#### CD4^+^ T Cell Functional Subsets

Different CD4^+^ T cell subsets that are generated from T_N_ were shown to be HIV-1 infected. For instance, resting CD4^+^CD25^+^ T_reg_ were found to be sensitive to HIV-1 infection acting as a viral reservoir in patients under long ART (Tran et al., 2008). These cells are characterized by a lesser response to T cell activation which limits virus expression and inhibits CD8^+^ T-cell cytotoxic function (Tran et al., 2008; Pardons et al., 2019). Additionally, the gamma-delta T cells (γδ T) which represent approximately 2 to 10% of total circulating CD3^+^ T lymphocytes and whose majority express TCR from Vδ2 variable regions (hereafter referred as Vδ2 cells), are classified as memory cells according to the expression of CD45RO and CD27 markers (Miyawaki et al., 1990). These Vδ2 cells have been documented to be productively infected and depleted upon HIV-1 infection (Li and Pauza, 2011). More recently, it has been demonstrated that Vδ2 cells in ART-treated patients with complete suppression of HIV-1 plasma viremia harbor latent HIV-1 that can replicate following *ex vivo* stimulation indicating that peripheral Vδ2 T cells are a potential HIV-1 reservoir (Soriano-Sarabia et al., 2015). Also, Th17 CCR6^+^ memory CD4^+^ T-cell subsets in the blood and colon are long-lived cells that act as HIV-1 reservoirs during ART (Gosselin et al., 2010, 2017; Pardons et al., 2019). In addition, T follicular helper cells (T_fh_) from the germinal center and peripheral blood (pT_fh_) are highly susceptible to HIV-1 infection holding replication-competent virus and serve as reservoirs during ART (Perreau et al., 2013; Pallikkuth et al., 2015; Kohler et al., 2016; Pardons et al., 2019). These cells are characterized by surface expression of CXCR5 and PD-1, reside in the lymph node follicles in immediate anatomical proximity to B cells, and support the germinal center reaction essential for the generation of effective humoral immunity. Notably, the group of Matthieu Perreau, by investigating lymph node T_fh_ (expressing CXCR5 and PD-1) and pT_fh_ (expressing CXCR3), has shown that these subpopulations are the major sources of infectious replication-competent HIV-1 (Banga et al., 2016b, 2018).

Very recently, resident memory CD4^+^ T cells (T_RM_), present in tissues such as the lower female genital tract has been described as a critical HIV-1 reservoir in cervical mucosa (Cantero-Pérez et al., 2019). Interestingly, cervical tissues from aviremic ART-treated HIV-1 infected woman contained higher viral DNA content compared to blood samples and showed that CD4^+^ T_RM_ harboring viral DNA and viral RNA are the main contributors to this reservoir.

#### Markers of Latently-Infected CD4^+^ T Cells

Studies investigating the role in latency of activation markers such as HLA-DR and immune checkpoint molecules (i.e., PD-1, LAG-3, TIGIT and Tim-3) have indicated that these markers are preferentially expressed at the surface of memory CD4^+^ T cells (T_CM_ and T_TM_) harboring latent HIV-1 provirus (Fromentin et al., 2016; Evans et al., 2018; Pardons et al., 2019). Although several studies, including those carried on SIV-infected macaques, have demonstrated that cells expressing these markers carry latent, replication-competent integrated viral DNA (Chomont et al., 2009; Hurst et al., 2015; Banga et al., 2016b; Fromentin et al., 2016; McGary et al., 2017), the replication competence of the integrated proviruses and the contribution of the cells bearing these markers to the latent reservoir still need to be fully elucidated.

Recently, the expression of CD32a has been reported as a potential marker of memory CD4^+^ T cells harboring a replication-competent latent virus in aviremic patients under ART (Descours et al., 2017; Darcis et al., 2019). The role of CD32a as a cellular marker of HIV-1 reservoirs has been the subject of several works (Abdel-Mohsen et al., 2018; Martin et al., 2018; Osuna et al., 2018; Thornhill et al., 2019). A complete study presented at CROI by Darcis et al. (CROI 2019, Poster 346 - CD32^+^ CD4^+^ T cells are enriched in HIV-1 DNA) showed that active CD4^+^ T cells co-expressing HLA-DR and CD32a are highly enriched with HIV-1 DNA.

The integrin α4β7 has been shown on a T cell subset that is highly susceptible to HIV-1 infection (Cicala et al., 2009; Sivro et al., 2018). Moreover, the integrin α4β1 was shown to be expressed by more than 70% of infected cells both in untreated and ART-suppressed individuals (Pardons et al., 2019). Integrins mediate the adhesion and transendothelial migration of lymphocytes facilitating their homing to GALT (for α4β7) and to the inflamed central nervous system/bone marrow (for α4β1) suggesting a role in HIV-1 persistence by enhancing T cell expansion.

In addition, high levels of CD2 receptor expression on latently infected resting memory CD4^+^ T cells in virally suppressed HIV-1-infected subjects has been also identified (Iglesias-Ussel et al., 2013). Moreover, CD30 receptor was identified on transcriptionally active CD4^+^ lymphocytes in individuals on suppressive ART suggesting that it might be a marker of residual, transcriptionally active HIV-1 infected cells in the setting of suppressive ART (Hogan et al., 2018). Last but not least, very recently the B lymphocyte antigen CD20 has also been identified as a marker of transcriptionally-active HIV-infected cells (Serra-Peinado et al., 2019).

### Non-T Cell Reservoirs

Even though memory CD4^+^ T cells are a long-term cellular reservoir for HIV-1, they are not the only source of viral rebound during treatment interruption. Macrophages, DCs, and tissue macrophages, such as microglial cells, are part of the viral reservoir (Kumar et al., 2014; Kandathil et al., 2016; Honeycutt et al., 2017; Schwartz et al., 2019).

Indeed, overwhelming evidence supports the notion that tissues, such as the CNS (Canestri et al., 2010; Gras and Kaul, 2010), lymph nodes (Sewald et al., 2015), testes (Darcis et al., 2016; Jenabian et al., 2016), gut (Yukl et al., 2013), genital tract (Iversen et al., 2004; Marcelin et al., 2008; Sheth et al., 2009), and lungs (Costiniuk and Jenabian, 2014), serve as HIV-1 sanctuaries that counteract viral eradication. In this regard, the group of Morgane Bomsel has recently reported that urethral tissue macrophages expressing IL-1 receptor, CD206, and IL-4 receptor, but not CD163, constitute a replication-competent HIV-1 reservoir (Ganor et al., 2019). Importantly, lipopolysaccharides specifically reactivate the production of replication-competent infectious proviruses from these tissue macrophages (Ganor et al., 2019).

In the case of circulating monocytes, they are more resistant to HIV-1 infection and their contribution as a viral reservoir is controversial and remains debatable. Monocytes have been proposed as a vehicle of HIV-1 dissemination throughout the body upon their entry in tissues where they differentiate into macrophages. Especially, due to their ability to cross the blood-tissue barrier, monocytes consequently, spread the infection into sanctuaries such as the brain (Pulliam et al., 1997; Valcour et al., 2009, 2010, 2013; Williams et al., 2014). These characteristics indicated the monocytes as an important viral reservoir. However, even if several studies have reported persistent infection of monocytes in ART treated individuals (Lambotte et al., 2000; Sonza et al., 2001; Gibellini et al., 2008), others failed to detect HIV-1 in circulating monocytes (Almodóvar et al., 2007). A recent work from the group of Nicolas Chomont indicated that monocyte infection is infrequent, and they highlighted the importance of using flow cytometry cell-sorting to minimize contamination by CD4^+^ T cells (Massanella et al., 2019).

Follicular dendritic cells (fDCs) in lymphoid tissues specialized in the trapping and retention of antigens in the form of immune complexes on their surface, including HIV-1 virions, can serve as a potential viral reservoir (Smith et al., 2001). Using specific and sensitive next-generation *in situ* hybridization approach, Deleage et al. (2016) documented the importance of B cell follicles in active, latent, and persistent infections by analyzing lymphoid tissues from macaques prior to and during ART. These fDCs could thus transfer the virus to T cells present in the follicles of secondary lymphoid organs (Heesters et al., 2015). In addition, myeloid dendritic cells (mDCs) located in the lymph nodes may support a very low level of viral replication and have a role in HIV-1 latency (Shen et al., 2014). However, the mechanism of viral persistence in these cells is not yet clearly understood [reviewed in Kandathil et al. (2016)].

Viral DNA has also been detected in hematopoietic stem cells (HSCs) from ART-treated patients, which could demonstrate their involvement in HIV-1 persistence (Sebastian et al., 2017; Zaikos et al., 2018). Despite the limited infection and detection of HSCs in a subset of patients (McNamara et al., 2013), their role as a viral reservoir may be crucial. Finally, HIV-1 can infect kidney allografts after transplantation despite undetectable viremia thereby suggesting that podocytes can serve as viral reservoirs and revealing once again the heterogeneous composition of the HIV-1 reservoir (Canaud et al., 2014).

In conclusion, multiple cell subsets being present at various cellular differentiation states, either resting or activated, can serve as HIV-1 reservoir. All these cells contribute to the complexity and heterogeneity of the reservoirs of latent HIV-1. It is very likely that different molecular mechanisms of latency establishment and persistence are involved in different latently-infected cell types and even may vary among cells of the same lineage.

## Complexity of Molecular Mechanisms Regulating HIV-1 Latency

**FIGURE 1:**
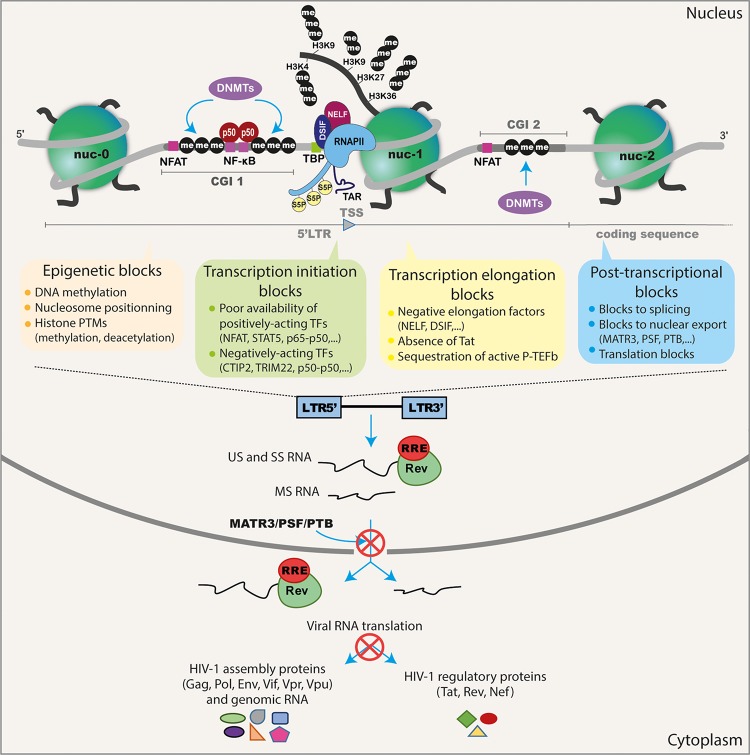
Schematic representation of the different transcriptional and post-transcriptional blocks involved in HIV-1 latency. During HIV-1 latency, several blocks preventing viral production have been described. These are represented by the methylation of the two CGIs surrounding the HIV-1 TSS and the deposit of repressive epigenetic marks (histone deacetylation and methylation) maintaining the repressive nucleosome nuc-1 positioned in the HIV-1 5′LTR promoter just downstream the TSS. The transcription initiation is also blocked because of the cytoplasmic sequestration of the positive NF-κB heterodimer p50–p65 and the phosphorylated NFAT and STAT5. The presence of repressive factors (such as CTIP2, TRIM22 and the binding of the homodimer p50–p50 to the NF-κB binding sites in the HIV-1 promoter) acts negatively on HIV-1 transcription initiation. The RNAPII, with its phosphorylated serine 5 (S5P) residue in its C-terminal domain, pauses and accumulates at the promoter-proximal region due to the binding of the negative factors NELF and DSIF. The elongation is also blocked by the absence of the master regulator of viral transcription Tat and by the sequestration of the positive transcriptional elongation factor P-TEFb into the inactive complex named 7SK snRNP. The splicing and export of HIV-1 transcripts are inefficient during latency due to the low expression level of post-transcriptional factors such as PTB, MATR3, and PSF. Finally, translation of viral transcripts could be inhibited by mechanisms involving mRNA degradation and sequestration in cytoplasmic granules.

It is well established that repression of HIV-1 expression is mediated through a plethora of molecular mechanisms both for the establishment and for the maintenance of post-integration latency (summarized in Figure 1). In fact, HIV-1 latency is a multifactorial phenomenon, controlled by several interlinked mechanisms operating at the transcriptional and post-transcriptional levels [reviewed in Van Lint et al., 2013)] and influenced by the transcriptional program of the host cell (Bradley et al., 2018). For instance, some studies have suggested that HIV-1 viral latency is related to the integration site and orientation of the provirus (Greger et al., 1998; Han et al., 2008; Lenasi et al., 2008; Chen et al., 2017; Einkauf et al., 2019). HIV-1 integration seems random but favors the introns of transcriptionally-active genes located in gene dense regions in the outer shell of the nucleus close to the nuclear pores (Schröder et al., 2002; Han et al., 2004; Marini et al., 2015; Singh et al., 2015; Chen et al., 2017). The importance of transcriptional interference as a mechanism suppressing the expression of the integrated provirus (including steric hindrance, convergent transcription, and enhancer trapping) has also been proposed (Greger et al., 1998; Han et al., 2008; Lenasi et al., 2008) [reviewed in Colin and Van Lint (2009)].

Moreover, during latency, the HIV-1 promoter is heavily controlled by epigenetic mechanisms, including DNA methylation (Blazkova et al., 2009; Kauder et al., 2009; Chávez et al., 2011) and histone post-translational modifications, such as histone acetylation (Lusic et al., 2003; Jiang et al., 2007; Tyagi and Karn, 2007; Li et al., 2018), methylation (du Chéné et al., 2007; Marban et al., 2007; Imai et al., 2010; Friedman et al., 2011; Ding et al., 2013; Tchasovnikarova et al., 2015, 2017; Boehm et al., 2017; Nguyen et al., 2017; Zhang et al., 2017; Huang et al., 2019), and crotonylation (Jiang et al., 2018). The degree of DNA methylation on the HIV-1 promoter was long considered controversial due to conflicting observations in patients (Blazkova et al., 2009, 2012; Kauder et al., 2009; Ho et al., 2013; Weber et al., 2014). However, this heterogeneity in promoter methylation profile between patients has now been explained by the duration of the infection (Palacios et al., 2012) and/or the duration of ART therapy (Trejbalová et al., 2016) which impacts the accumulation of cytosine methylation on the 5′LTR. Still, the exact mechanisms of this DNA methylation accumulation in the latent reservoir of HIV-1-infected individuals remain unclear.

Additionally, an antisense transcript originating from the 3′LTR (named the *ASP* RNA) (Zapata et al., 2017) was recently shown to recruit the PRC2 repressor complex to the HIV-1 5′LTR, increasing the repressive epigenetic mark H3K27me3 while reducing RNAPII occupancy at the viral promoter and promoting the establishment and maintenance of HIV-1 latency at the epigenetic level (Zapata et al., 2017). An HIV-encoded antisense long non-coding RNA directs the same epigenetic silencing mechanisms by recruiting and guiding chromatin-remodeling complex consisting of proteins such as DNMT3a, EZH2, and HDAC-1 to the viral promoter leading to transcriptional HIV-1 latency (Saayman et al., 2014).

HIV-1 latency is also associated with poor availability of transcriptional activating factors including NF-κB, NFAT, and STAT5 due to their cytoplasmic sequestration (Williams et al., 2006; Della Chiara et al., 2011; Hakre et al., 2012; Bosque et al., 2017) and with a low expression level of the viral transactivator Tat (Burnett et al., 2009; Weinberger et al., 2005). The interaction of the cellular transcriptional cofactor CTIP2 with HP1α is involved in the relocation of Tat to transcriptionally inactive regions of the chromatin (Rohr et al., 2003). However, HIV-1 counteracts CTIP2 mediated-repression by promoting its degradation by the HIV-1 accessory protein Vpr (Forouzanfar et al., 2019). Another recent finding has revealed that APOBEC3A, the restriction factor that suppresses HIV-1 infection in macrophages, maintains HIV-1 latency by recruiting KAP1 and HP1 inducing repressive histone marks (Taura et al., 2019). In addition, the importance of TRIM22, known to inhibit the binding of Sp1 to the 5′LTR promoter (Turrini et al., 2015) and to colocalize with HIV-1 transcriptional repressors TRIM19/PML (Forlani et al., 2017), contributes to the maintenance of HIV-1 latency (Turrini et al., 2019).

During latency and even after transcription initiation, the RNAPII is paused during early elongation, constraining its processivity and resulting in repression of HIV-1 gene expression (Adams et al., 1994). RNAPII terminates prematurely and accumulates at specific positions of the HIV-1 promoter that overlap with the position of the repressive nucleosome nuc-1, positioned in the 5′LTR just after the TAR region, inducing a block of transcriptional elongation (Verdin et al., 1993). This inhibition of HIV-1 transcription elongation is regulated by the binding of negative factors such as NELF and DSIF to the RNAPII (Yamaguchi et al., 1999; Jadlowsky et al., 2014) and by the limited availability of the cellular transcription factor P-TEFb due to its sequestration by the 7SK snRNP complex (Nguyen et al., 2001; Yang et al., 2001; Cherrier et al., 2013; Eilebrecht et al., 2014). P-TEFb is composed of CyclinT1 and CDK9 and requires the CDK9 Thr-186 phosphorylation for its activity to mediate the phosphorylation of the C-terminal domain of the paused RNAPII antagonizing its negatives factors (Wei et al., 1998; Ramakrishnan et al., 2009). The level of CDK9 phosphorylation has been found to be lower in resting CD4^+^ T cells harboring latent HIV-1 (Budhiraja et al., 2013; Ramakrishnan et al., 2015). Furthermore, CTIP2 binds to the 7SK snRNP complex to inhibit P-TEFb and HIV-1 transcription in microglial cells (Cherrier et al., 2013; Eilebrecht et al., 2014).

Abortive TAR transcripts have been reported even during transcriptional elongation block (Adams et al., 1994; Core and Lis, 2008; Kaiser et al., 2017). In a very elegant manner, the group of Steven Yukl has evidenced that multiple blocks to transcription and even to splicing and export exist in patient cells. They developed a panel of RT-ddPCR assays to measure at the same time different HIV-1 transcripts (Telwatte et al., 2018; Yukl et al., 2018) in patient-derived cells. They quantified the transcripts suggestive of transcriptional interference (U3-U5), initiation (TAR), 5′ elongation (R-U5-pre-Gag), distal transcription (Nef), completion (U3-polyA), and multiple splicing (Tat-Rev) (Yukl et al., 2018). Using blood CD4^+^ T cells from HIV-1 individuals under ART, they showed a greater block to HIV-1 transcriptional elongation, distal HIV-1 transcription, completion, and multiple splicing than to transcriptional initiation (Yukl et al., 2018). This transcription profiling approach highlighted the blocks at distinct stages of HIV-1 transcription and splicing (which may be governed by different mechanisms), thereby underlying the heterogeneity of HIV-1 latency in CD4^+^ T cells.

The work of Telwatte et al. (2018) and Yukl et al. (2018) and also of Pace et al. (2012) have highlighted the importance of post-transcriptional blocks in HIV-1 latency. Post-transcriptional blocks of the nuclear export of various viral transcripts including unspliced, partially spliced, multiply-spliced, and translation of HIV-1 RNA by miRNAs have also been reported (Pomerantz et al., 1990; Malim and Cullen, 1991; Lassen et al., 2006; Weng et al., 2014; Modai et al., 2019). Interestingly, a recent study from the group of Alessandro Marcello has demonstrated that the expression level of MATR3 and PSF, two known post-transcriptional cofactors of the HIV-1 protein Rev required for Rev-mediated export of RRE-containing HIV-1 RNAs (Kula et al., 2011, 2013), are poorly expressed in latently-infected patients cells (Sarracino et al., 2018), suggesting a novel post-transcriptional block linked to RNA export. This latter block was reversed by ectopic overexpression of MATR3 which boosted the action of the HDAC inhibitor SAHA in the J-Lat cell line model for HIV-1 latency (Sarracino et al., 2018). These results demonstrated the importance of post-transcriptional blocks, especially at the level of viral RNAs export, that need to be relieved to reach full viral reactivation by LRAs. Similarly, the work of Andrew Mouland and colleagues has demonstrated that UPF-1, a known RNA surveillance protein (Ajamian et al., 2015), acts as a positive post-transcriptional regulator of viral reactivation from latency (Rao et al., 2018).

Additional post-transcriptional mechanisms including the involvement of cellular miRNA and non-coding RNAs in latency have been described. More specifically, the regulation of viral expression and production by miRNAs targeting PCAF (Triboulet et al., 2007) and CyclinT1 (Sung and Rice, 2009; Budhiraja et al., 2013) have been related to latency (Huang et al., 2007). Many of these miRNAs involved in this process are expressed in resting cells but are downregulated during T-cell activation (Huang et al., 2007). For instance, the lower susceptibility to HIV-1 infection of monocytes in comparison to macrophages has been shown to be correlated with high and low expression of these miRNAs, respectively (Klase et al., 2007). Finally, lncRNA called NRON which is strongly expressed in resting CD4^+^ T cells was shown to be involved in HIV-1 latency by inducing Tat degradation through the proteasome pathway (Li et al., 2016). The combination of SAHA with NRON knockdown significantly reactivates viral production from latently-infected primary CD4^+^ T cells (Li et al., 2016).

Altogether, the complexity of the molecular mechanisms underlying HIV-1 latency marks the heterogeneous nature of the HIV-1 reservoirs.

## Shock and Kill Strategy to Eliminate the Latent Reservoir

Mechanistic insights into HIV-1 transcriptional repression during latency have allowed to develop the “shock and kill” strategy. This approach is based on the use of small-size chemical compounds called LRAs which reactivate HIV-1 from latency (the “shock” phase) while maintaining ART in order to prevent new infection events (Sengupta and Siliciano, 2018). This kind of strategy would allow the “kill” phase during which latently-infected cells would then die from viral cytopathic effects or host cytolytic effector mechanisms following viral reactivation. Of note, this strategy does not discriminate between replication-competent and defective proviruses. Several classes of LRAs have been developed and studied (reviewed in Kim et al., 2018; Spivak and Planelles, 2018; Sadowski and Hashemi, 2019). These include: PKC agonists, MAPK agonists, CCR5 antagonist, Tat vaccine, SMAC mimetics, inducers of P-TEFb release, activators of Akt pathway, benzotriazole derivatives, epigenetic modifiers (including HDACis, HMTis, and DNMTis), and immunomodulatory LRAs (including TLR agonists, IL-15 agonist and immune checkpoint inhibitors) (summarized in Table 1).

**TABLE 1:** Classes of HIV-1 latency reversing agents.

LRA classes	Examples	Targets	References
PKC agonists	Prostratin Bryostatin-1 Ingenols: Ingenol-B, Ingenol 3,20-dibenzoate (Ingenol-db), ingenol-3-angelate (ingenol mebutate, PEP005)	NF-κB activation	Kulkosky et al., 2001; DeChristopher et al., 2012; Jiang et al., 2014; Darcis et al., 2015; Spivak et al., 2015
MAPK agonist	Procyanidin trimer C1	MAP Kinase activation	Cary and Peterlin, 2018
CCR5 antagonist	Maraviroc	NF-κB activation	López-Huertas et al., 2017; Madrid-Elena et al., 2018
Tat vaccine	Tat Oyi vaccine Tat-R5M4 protein	Activation of HIV-1 LTR	Geng et al., 2016
SMAC mimetics	SBI-0637142 Birinapant	Induction of non-canonical NF-κB pathways	Pache et al., 2015; Hattori et al., 2018
Inducers of P-TEFb release	BETis: JQ1, I-BET, I-BET151, OTX015, UMB-136, MMQO, CPI-203, RVX-208, PFI-1, BI-2536 and BI-6727 HMBA	Release of P-TEFb	Contreras et al., 2007; Bartholomeeusen et al., 2012; Darcis et al., 2015; Lu et al., 2016; Huang et al., 2017; Lu et al., 2017; Abner et al., 2018; Gohda et al., 2018; Liang et al., 2019
Activators of Akt pathway	Disulfiram	Upregulation of Akt signaling pathway	Xing et al., 2011; Doyon et al., 2013; Spivak et al., 2014
Benzotriazole derivatives	1-hydroxybenzotriazol (HOBt)	STAT5 activation	Bosque et al., 2017
Epigenetic modifiers	HDACis: TSA, trapoxin, SAHA, romidepsin, panobinostat, entinostat, givinostat, valproic acid, MRK-1/11, AR-42, fimepinostat, chidamide	HDAC inhibition	Van Lint et al., 1996; Quivy et al., 2002; Ylisastigui et al., 2004; Archin et al., 2009; Archin et al., 2012; Rasmussen et al., 2013, 2014; Wightman et al., 2013; Mates et al., 2015; Søgaard et al., 2015; Banga et al., 2016a; Kuai et al., 2018; Gunst et al., 2019; Yang et al., 2018
	HMTis: chaetocin, EPZ-6438, GSK-343, DZNEP, BIX-01294, UNC-0638	Suv39H1, G9a, SMYD2	Friedman et al., 2011; Bouchat et al., 2012; Nguyen et al., 2017
	DNMTis: 5-AzaC, 5-AzadC	DNMT1, 3a, 3b	Bouchat et al., 2016
Immunomodulatory LRAs	TLR agonists: TLR2 (Pam3CSK4), TLR7 (GS-9620), TLR8, TLR9 (MGN 1703) agonists IL-15 agonist (ALT-803) Immune checkpoint inhibitors: anti-PD-1 (nivolumab, pembrolizumab), anti-CTLA-4 (ipilimumab)		Schlaepfer and Speck, 2011; Novis et al., 2013; Wightman et al., 2015; Jones et al., 2016; Offersen et al., 2016; Tsai et al., 2017; Evans et al., 2018

It has been further demonstrated by several groups, including ours, that targeting a single mechanism might not be efficient enough to reactivate the majority of latent proviruses and that combinations of LRAs acting on several HIV-1 silencing molecular mechanisms are needed to obtain synergistic viral reactivations and achieve a more significant decrease in the size of the reservoirs (Reuse et al., 2009; Bouchat et al., 2012, 2016; Darcis et al., 2015; Jiang et al., 2015; Laird et al., 2015; Pache et al., 2015; Tripathy et al., 2015; Abdel-Mohsen et al., 2016; Chen et al., 2017; Rochat et al., 2017; Das et al., 2018). Moreover, when LRAs are used in combination, lower concentrations are effective, thereby reducing the toxicity of each LRA. However, even if these LRA combinations reverse latency, they could inhibit multiple CD8^+^ T cell function [reviewed in Clutton and Jones (2018)]. Importantly, despite the potent effect of a single LRA and of LRA combinations *in vitro* and *ex vivo*, multiple clinical trials have failed to show a decrease in the size of the latent reservoir *in vivo*. A clear change in plasma HIV-1 RNA with a subsequent decrease in the reservoir size *in vivo* has been seen only for the immune check point inhibitor nivolumab (Guihot et al., 2018) and for romidepsin (combined with an immunovaccine) (Leth et al., 2016) but without prolonging time to viral rebound after ART interruption in this latter study (Leth et al., 2016). In the case of nivolumab, the study was conducted only on one patient who experienced a drastic and sustained decrease of the HIV-1 reservoir (Guihot et al., 2018). However, other controversial studies found no consistent changes in the size of the latent reservoir nor in HIV-specific CD8^+^ T-cell responses in HIV-1-infected individuals treated with anti-PD1 antibodies (including nivolumab) (Le Garff et al., 2017; Evans et al., 2018; Lavolé et al., 2018; Scully et al., 2018). Success to completely eradicate latent viruses with LRAs is hampered by the heterogeneous nature of the latent reservoir and by the diversity of the silencing mechanisms governing latency that make the current “shock and kill” strategy inefficient.

## Latency Reversing Agents Highlight the Heterogeneous Nature of the Latent Reservoir

All studies investigating LRAs have demonstrated the heterogeneous nature of the cellular and tissue reservoirs of latent HIV-1 and their diverse reactivation capacity, highlighting the different determinant responsible for the heterogeneous responses to LRAs. All these studies are summarized in Table 2.

**TABLE 2:** The diverse responses of latently-infected cells to LRAs reflect the heterogeneity of the mechanisms driving HIV-1 latency.

Heterogeneity determinants	References	Cellular or tissue reservoir	Methodology	Heterogeneous responses to LRAs	
				LRAs	Results illustrating heterogeneous responses to LRAs
Virus genetic background	Norton et al., 2019	Jurkat cells infected with HIV-1 WT or mutated in ESE_tat_.	Flow cytometry	PMA JQ1 Panobinostat	Mutations altering viral gene splicing (tat mRNA) lead to more silent phenotypes that are differently reactivated by diverse LRAs.
Cell model	Spina et al., 2013	J-Lat 6.3, 8.4, 11.1 and 5A8. Primary T-cell models of HIV-1 latency. *Ex vivo* T-cell cultures from HIV-1^+^ individuals.	Flow cytometry QVOA	Anti-CD3 + anti-CD28, PHA, PMA, prostratin, bryostatin, PMA + ionomycin, TNFα, IL-7 + IL-2, SAHA, MRK-1, MRK-11, HMBA, ionomycin	None of the *in vitro* T cell model alone is able to capture accurately the *ex vivo* response characteristics of latently-infected T cells isolated from HIV^+^ individuals.
Cell type	Grau-Expósito et al., 2019	Patient-derived diverse subsets of memory CD4^+^ T cells.	Flow-based RNA FISH	Romidepsin Panobinostat JQ1 Ingenol-3-angelate Bryostain-1	Romidepsin acts on majority of the T-cell subsets (T_CM_, T_EM_, T_TM_, and T_NA_) except for T_SCM_. Ingenol reactivates majority of T-cell subsets (T_NA_, T_SCM_, T_CM_, and T_TM_) except for T_EM_. Panobinostat acts mainly on T_CM_ and slightly on T_EM_ and T_NA_. Bryostatin-1 reactivates very modestly T_NA_, T_TD_, and T_CM_. JQ1 acts very modestly on the majority of the subsets, except for T_SCM_. Romidepsin + ingenol is the most potent combination generating p24 only in T_CM_.
	Kulpa et al., 2019	*Ex vivo* T-cell cultures from HIV-1^+^ individuals and *in vitro* model of HIV-1 latency LARA.	Flow cytometry Cell sorting TILDA	Bryostatin IL-15 PMA + ionomycin	T_CM_ cells differentiate into T_EM_ cells when exposed to LRAs. The increase of T_EM_ subset frequencies is predictive of higher prevalence of cells carrying an inducible reservoir.
	Baxter et al., 2016	Diverse subsets of patient-derived CD4^+^ T cells.	Flow-based RNA FISH	Bryostatin-1 Ingenol-3-angelate	Bryostatin-1 mainly reactivates T_EM_. Ingenol reactivates T_CM_, T_TM_ and T_EM_.
	Kula et al., 2019	U1, THP89, CHME5 and J-Lat 9.2, J-Lat A1 and A2.	Flow cytometry	Disulfiram	Disulfiram reactivates HIV-1 in 3 myeloid infected cell lines but not in the infected T-lymphoid cell lines.
Latency molecular mechanisms	Yukl et al., 2018	Patient-derived blood CD4^+^ T cells.	RT-ddPCR	Panobinostat Romidepsin Ingenol mebutate	Panobinostat and romidepsin increase full-length and elongated transcripts, while ingenol mebutate increases polyadenylated and multiply spliced transcripts.
Tissue reservoir	Elliott et al., 2014	Patient-derived blood and rectal CD4^+^ T cells.	Semi-nested RT-qPCR	SAHA	Fold change in CA-US HIV-1 RNA following SAHA is 5 times higher in CD4^+^ T cells from blood compared to rectal tissue from HIV-1^+^ individuals.
Integration site of the provirus and chromatin context	Chen et al., 2017	Jurkat cells infected with B-HIVE.	Sorting of the GFP^+^ cells coupled with inverse PCR and provirus mapping	PHA SAHA	PHA and SAHA reactivate proviruses located at distinct integration sites but with an increased frequency in the proximity of enhancers for SAHA.
	Abner et al., 2018	Jurkat cells infected with B-HIVE.	Sorting of the GFP^+^ cells coupled with RT-qPCR and provirus mapping	MMQO JQ1 SAHA Prostratin	BETi (MMQO and JQ1) target viruses integrated at distinct sites as compared to those targeted by SAHA and prostratin.
	Battivelli et al., 2018	Primary CD4^+^ T cells infected with dual-labeled HIV-1.	Cells sorting coupled with semi-nested ligation-mediated PCR and provirus sequencing	Panobinostat JQ1 Bryostatin-1 Anti-CD3 + anti-CD28	LRAs reactivate only 5% of latently-infected cells. The inducible and non-inducible populations exhibit distinct chromatin integration sites which were associated, respectively, with active chromatin and heterochromatin with non-accessible region.
Patient to patient and patient gender	Das et al., 2018	Patient-derived resting memory CD4^+^ T cells.	EDITS	Anti-CD3 + anti-CD28 SAHA	Women have reduced inducible RNA reservoirs compared to men following treatment with anti-CD3 + anti-CD28. ESR-1 antagonists potentiate HIV-1 reactivation by SAHA, however, females show higher reactivation than males HIV-1^+^ individuals.
	Darcis et al., 2017	Patient-derived CD8^+^-depleted PBMCs and resting CD4^+^ T cells.	Highly sensitive TaqMan based RT-qPCR	JQ1 + bryostatin JQ1 + ingenol-B 5-AzadC + panobinostat 5-AzadC + romidepsin	There is a positive correlation between the HIV-1 reservoir size and the *ex vivo* capacity of HIV-infected patient cell cultures to be reactivated by LRAs. However, some HIV-1^+^ patients deviate from this linearity (for example, patients who, despite a low reservoir, are more easily reactivated than many other patients who have a larger reservoir).
	Yukl et al., 2018	Patient-derived CD4^+^ T cells.	RT-ddPCR	JQ1, Disulfiram Chaetocin, Panobinostat Romidepsin, Ingenol mebutate, Ingenol 3,20-dibenzoate	All LRAs exhibit inter-patient variability to reverse the blocks to HIV-1 transcription with a very weak exception for romidepsin.

*B-HIVE, barcoded HIV ensembles; CA-US RNA, cell-associated unspliced RNA; EDITS, envelope detection by induced transcription-based sequencing; LARA, latency and reversion assay; RNA FISH, RNA Fluorescence *In Situ* Hybridization; RT-ddPCR, reverse transcription droplet digital polymerase chain reaction; TILDA, Tat/rev-induced limiting dilution assay; QVOA, Quantitative Viral Outgrowth Assays.*

### HIV-1 Diversity Within the Latent and Reactivated Reservoirs

Few studies so far have focused on investigating the contribution of the viral diversity, the compartmentalization, the intact or defective nature of the viral reservoir, and the origin of the rebounding virus in latency reversal. A previous study in patients who initiated ART during acute infection showed that proviral sequences from PBMCs and GALT presented low level of genomic diversity and divergence and remained unchanged after treatment interruption (Lerner et al., 2011). Moreover, there was no phylogenetic link between the rebounded plasma viral sequences and those from the GALT proviral DNA, indicating that HIV-1 cellular reservoirs in the GALT may be different from those circulating in peripheral blood and might not contribute to the rebounded plasma viremia (Lerner et al., 2011). Other studies have supported the compartmentalization idea of the viral population in the gut with divergent opinions (van der Hoek et al., 1998; Lewis et al., 2013; Rozera et al., 2014). Depending on the stage of HIV-1 infection, the diversity of HIV-1 RNA appears lower in patients with early infection *versus* chronic infection, and thus, compartmentalization is lost during chronic infection (Rozera et al., 2014). However, other works have supported other conflicting ideas with findings showing absence of compartmentalization of HIV-1 between the gut and blood (Avettand-Fenoel et al., 2011; Imamichi et al., 2011; Evering et al., 2012), providing evidence for cross infection between these two compartments (Chun et al., 2008).

HIV-1 sequence diversity has been reported to be either higher (Klein et al., 2018) or similar (Piantadosi et al., 2019) to genital tract compared to blood. Viral compartmentalization between the blood and the male genital tract has been reported by multiple studies including SIV-infected macaques (Delwart et al., 1998; Paranjpe et al., 2002; Pillai et al., 2005; Coombs et al., 2006; Diem et al., 2008; Houzet et al., 2018). More recently, patients under suppressive ART exhibited a significant positive correlation between viral diversity and genetic compartmentalization in the blood and testes, but it was attributable to differential frequencies of identical HIV-1 sequences between the two sites (Miller et al., 2019). However, there was no evidence of compartmentalization when only unique sequences per sites are considered, suggesting that compartmentalization between blood and testes is linked to clonal expansion (Miller et al., 2019).

HIV-1 phylogenetic analysis of *post-mortem* CSF, brain, and spleen from HIV-1 patients under ART and presenting dementia symptoms showed that HIV-1 strains from the blood and spleen are different from those in the brain and CSF (Caragounis et al., 2008). A more recent study of *env* and *nef* phylogeny on ART suppressed individuals confirmed the presence of considerable viral diversity in the spleen and lymph nodes (Nolan et al., 2018). However, there is no viral compartmentalization between spleen and PBMCs in SIV-infected and suppressed rhesus macaques (Siddiqui et al., 2019).

The comparison of latent HIV-1 from the blood and lymph node CD4^+^ T cells from HIV-1 individuals undergoing ART interruption, after TLR9 coadministration with ART, suggests the same frequencies of intact proviruses in the blood and lymph nodes and the fact that CD4^+^ T cells carrying latent viruses circulate between the blood and lymphoid tissues. However, there is no overlap between latent reservoirs and rebounded virus, thereby supporting the idea that recombinations may play a role in the emergence of the rebounded viremia (Vibholm et al., 2019).

Time to ART from estimated date of infection as an early ART initiation is associated with less molecular diversity in CSF without impacting HIV-1 DNA provirus compartmentalization in the CNS which occurs very early after infection (Schnell et al., 2010; Sturdevant et al., 2015; Oliveira et al., 2017). In the study of Oliveira et al. (2017), a higher diversity in PBMCs than in CSF is reported. The compartmentalized HIV-1 RNA in CNS is found to contribute to viral rebound within the CSF in patients undergoing treatment interruption but is phylogenetically distinct from those present in the paired blood plasma (Gianella et al., 2016).

Most of the previous studies estimated that blood memory CD4^+^ T cells are the source of viral rebound after ART interruption or viral reactivation from latency. The first work of Timothy Schacker and colleagues examined HIV-1 variants by single-genome amplification and phylogenetic analyses in matched lymph nodes, GALT biopsies and blood from HIV-1 suppressed individuals under longstanding ART after treatment interruption (Rothenberger et al., 2015). The rebounded virus after treatment interruption was found to be detectable in latently-infected cells at multiple sites with a highly complex and genetically diverse population of virions which bring out the challenges facing the heterogeneity of HIV-1 reservoir (Rothenberger et al., 2015). Another study of the effect of a brief treatment interruption on the HIV-1 latent reservoir of individuals who initiated ART during chronic infection showed no alteration either in the size or in the diversity of the peripheral reservoir and highlighted the substantial variability and the prevalence of clonally-expanded viral populations (Salantes et al., 2018). Interestingly, the group of Sarah Palmer indicated that analytical treatment interruption (ATI) activated proviruses with similar sequence between plasma and intestinal lamina propria mononuclear cells (Barton et al., 2016), indicating that intestinal HIV-1 reservoir is contributing to viremia following ATI. A recent study by the group of Linos Vandekerckhove confirmed the heterogeneous source of viral rebound from distinct anatomical reservoirs in HIV-1 individuals undergoing treatment interruption, showing that genetically-identical viral expansions play a significant role in viral rebound (De Scheerder et al., 2019). In a study presented by Oliveira et al. (CROI 2019, Poster 327—Characterizing the HIV DNA reservoirs in whole-body tissues in the “Last Gift” cohort), HIV-1 DNA was detected in most body tissues with a nice distribution and compartmentalization of HIV-1 reservoir between tissues from the Last Gift cohort enrolling altruistic, terminally-ill persons living with HIV-1. They have successfully sequenced *env* from 10 different tissues with many identical HIV-*env* sequences sampled in multiple body tissues.

Some LRA interventions analyzed whether the increase of CA-US HIV-1 RNA is related to limited or to broad activation of HIV-1 proviruses. The single genome sequencing of viral RNA transcripts showed that panobinostat and vorinostat activate genetically diverse HIV-1 proviruses that are similar to that observed during ATI but with a high percentage of defective viral sequences (Barton et al., 2016). More precisely, T_CM_ contributed to the rebounded viremia, indicating once again the important role of this subset in the persistence of latent HIV-1 (Barton et al., 2016). Finally, romidepsin administration to HIV-1 individuals under suppressive ART activates transcription from blood CD4^+^ T cells latent HIV-1 proviruses (Winckelmann et al., 2017). Importantly, the viremia induced by romidepsin contained few defective mutations and is characterized by low genetic diversity (Winckelmann et al., 2017, 2018).

Together, these studies illustrate that cure strategies should consider the complex and variable composition of the different viral reservoirs, the replication-competent capacity, the diversity and compartmentalization of HIV-1 reservoir, and the role of cellular clonal expansion and cellular proliferation in promoting HIV-1 persistence.

### Weak Reactivation Potential of Latency Reversing Agents and Post-transcriptional Blocks

An important issue for the development of a more effective “shock and kill” approach is to determine how effective LRAs are in terms of full viral reactivation from all latently-infected cells composing the reservoirs. Indeed, most of the *in vivo* reactivation studies demonstrated a rather weak reactivation effect of LRAs, i.e., an effect only on HIV-1 transcription with much less or no effect on plasma HIV-1 RNA (Archin et al., 2012; Spivak et al., 2014; Elliott et al., 2015). For instance, a recent clinical trial using escalating doses of disulfiram demonstrated that even if all doses produced an increase in the level of intracellular HIV-1 RNA, only the highest dose increased plasma HIV-1 RNA level albeit with very low effect (Elliott et al., 2015). Similar observations have been reported for SAHA: i.e., weak effect at the level of viral particle production *in vivo* as assessed by measurement of plasma HIV-1 RNA (Archin et al., 2012). Mohammadi et al. (2014) have shown in their primary CD4^+^ T cell models that disulfiram and SAHA treatments increased viral transcription, but failed to effectively enhance viral translation. In addition, the group of Maria Buzon has very recently demonstrated that a median of 16.28% of the whole HIV-reservoir exhibited HIV-1 transcripts induction after viral reactivation using various LRAs and their combinations, but only 10.10% of these HIV-1 RNA^+^ cells produced viral p24 proteins (Grau-Expósito et al., 2019). Recent work by the group of Andrew Lever (Norton et al., 2019) has demonstrated that inefficient splicing regulation may also influence the action of LRAs. They studied the polymorphisms occurring in a recently identified viral mRNA splicing regulatory element (ESE_tat_) regulating *tat* mRNA splicing which results in more silent phenotypes of the virus. Indeed, higher doses of LRAs (PMA, JQ1, and panobinostat) were required to reactivate silent viruses bearing the polymorphisms in ESE_tat_, reflecting their lower rate of inducibility as compared to wild-type HIV-1 (Norton et al., 2019). Therefore, different post-transcriptional mechanisms including blocks to export and alteration of mRNA splicing may be considered as druggable targets for a combined approach of more potent latency reversal (Sarracino et al., 2018; Norton et al., 2019).

### Cell Model-Specific Effects of Latency Reversing Agents

Latency reversing agents were shown to be cell model-specific exhibiting diverse reactivation profiles across multiple HIV-1 latency model systems. A comprehensive study by Spina et al. (2013) tested the potency of a panel of thirteen LRAs for their ability to reactivate HIV-1 in several broadly used HIV-1 latency models (primary T-cell models, multiple J-Lat cell lines, and *ex vivo* T-cell cultures derived from the blood of HIV-1^+^ individuals) (Spina et al., 2013). They showed that PHA was the only stimulus that uniformly reactivated latent HIV-1 in all these cell models, although other LRAs exerted largely heterogeneous responses among the various models (Spina et al., 2013). Importantly, following LRA treatment, none of the *in vitro* cell model systems could accurately capture the *ex vivo* response characteristics of latently-infected T cells from patients.

### Cell Type-Specific Effects of Latency Reversing Agents

In addition to cell model-specific effects of LRAs, recent studies demonstrated the cell type-specific effects of LRAs. For instance, Baxter et al. (2016) highlighted heterogeneous responses of CD4^+^ populations to bryostatin and ingenol. The authors showed that bryostatin induced HIV-1 expression in T_EM_ cells but had limited effect in T_CM_ and T_TM_ cells. While ingenol, on the other hand, exhibited more similar reactivation effects among the different memory T-cell subpopulations (Baxter et al., 2016). Similarly, the group of Maria Buzon has very recently demonstrated heterogeneous responses to LRAs of the latent reservoirs present in different CD4^+^ T-cell subpopulations (Grau-Expósito et al., 2019). Romidepsin and ingenol and their combination were the most potent LRAs at reactivating HIV-1 in almost all the subsets of CD4^+^ T cells by increasing, respectively, the proportion of T_CM_, T_EM_, T_TM_, T_NA_, and T_NA_, T_SCM_, T_CM_, T_TM_ cells expressing HIV-1 RNA. Panobinostat successfully reactivated HIV-1 only in T_CM_ cells. Bryostatin-1 reactivated very modestly some T-cell subsets, including T_NA_, T_TD_, and T_CM_. Therefore, T-cell differentiation status may impact the action of LRAs. Indeed, Kulpa et al. (2019) show in *ex vivo* and *in vitro* models that differentiated phenotype of T_EM_ cells from that of quiescent T_CM_ cells is associated with a potentiated response to LRAs and to a highest level of inducible HIV-1 reservoir. Additionally, the effects may also be cell type-specific. We have also recently demonstrated that disulfiram exhibited limited reactivation spectra, being active only in myeloid-derived HIV-1 latently infected cell lines (U1, THP89GFP monocytic, and CHME-5/HIV-1 microglial cells) but not in Jurkat-based T-cell lines (Kula et al., 2019). These heterogeneous cellular responses to LRAs indicate that distinct and cell-type dependent molecular mechanisms contribute to HIV-1 latency in diverse reservoirs.

### Latency Molecular Mechanism-Specific and Tissue-Specific Effects of Latency Reversing Agent

It has recently been demonstrated by Yukl et al. (2018), using an elegant transcriptional profiling approach, that LRAs exhibit silencing mechanisms-specific effects. These authors found that HDACis (panobinostat and romidepsin) and PKC agonists (ingenol 3,20-dibenzoate and ingenol mebutate) exert differential effects on the latency blocks in the blood latently-infected CD4^+^ T cells (Yukl et al., 2018). More specifically, HDACis increased total and elongated transcripts but had less or no effect on polyadenylated and multiply spliced transcripts, whereas ingenol mebutate strongly induced polyadenylated and multiply spliced transcripts but had lesser effects on transcription initiation and elongation (Yukl et al., 2018). These latter results are in agreement with another study showing that romidepsin administration after six doses of the therapeutic vaccine Vacc-4x, in HIV-1 individual under suppressive ART, increased early events in HIV-1 transcription (initiation and elongation) but had less effect on later stages (completion, multiple splicing) (Moron-Lopez et al., 2019). The differential effects of these LRAs suggest that the mechanisms underlying the blocks to completion and splicing may differ from those that mediate the blocks to initiation and elongation. The group of Yukl compared CD4^+^ T cells from the blood and rectum tissue reservoirs using a similar transcriptional profiling approach and found a much greater block to HIV-1 transcription initiation in the rectum compared to blood (Telwatte et al., 2018). Indeed, the ratio of total to elongated transcripts was 6-fold lower in the rectum CD4^+^ T cells, suggesting less of a block to HIV-1 transcriptional elongation in rectal CD4^+^ T cells (Telwatte et al., 2018). In fact, a multi-dose trial of SAHA has evidenced that the cell-associated HIV-1 RNA in latently infected CD4^+^ T cells from the blood was 5-fold higher compared to CD4^+^ T cells from the rectal tissue (Elliott et al., 2014), suggesting that the LRA-driven effects may also be tissue-specific. Thus, further studies should investigate whether gut cells differ from blood cells in their response to LRAs in terms of HIV-1 transcript production and of cellular gene expression.

### Integration Site-Specific Effects of Latency Reversing Agents

The group of Guillaume Fillion demonstrated that different LRAs reactivate different subsets of latent proviruses (Chen et al., 2018). Using a method called B-HIVE to map the chromosomal locations of individual proviruses, these authors revealed in Jurkat cells that responses to LRAs are also viral integration site-specific. They found that PHA and SAHA reactivated proviruses inserted at distinct genomic locations, suggesting that the insertion context of HIV-1 is a critical determinant of the viral response to LRAs (Chen et al., 2018). Using the same B-HIVE technology, the groups of Albert Jordan and Guillaume Fillion demonstrated that MMQO (an LRA which acts as an I-BET) and JQ1 reactivate latent HIV-1 proviruses integrated at distinct sites from those proviruses targeted by SAHA and prostratin (Abner et al., 2018). Interestingly, Battivelli et al. (2018) demonstrated that only less than 5% of latently-infected primary CD4^+^ T cells are reactivated by LRAs. By further sequencing analysis, these authors showed the preference toward integration events in active chromatin sites for the reactivable cell population, while these regions were significantly disfavored in the non-reactivable group, highlighting that the role of chromatin environment is an important determinant of LRA effectiveness (Battivelli et al., 2018).

### Patient-Specific and Sex-Specific Effects of Latency Reversing Agents

Many other determinants may also be responsible for the heterogeneous reactivation profile of LRAs. Patient-dependent effects of LRAs were reported. Indeed, viral productions evident in some patients but not in others were observed in all reactivation clinical trials (Spivak et al., 2014; Elliott et al., 2015; Leth et al., 2016; Tapia et al., 2017). Additionally, in our previous reactivation studies, we demonstrated patient-specific variations in terms of reactivation capacity of their *ex vivo* cell cultures following treatments with various LRAs (Darcis et al., 2015, 2017; Bouchat et al., 2016). Indeed, we established a positive correlation between the size of the HIV-1 reservoirs and the *ex vivo* capacity of HIV-1-infected patients’ cell cultures to be reactivated by LRAs (Darcis et al., 2017), but we identified HIV-1^+^ patients who deviated from this linearity relative to their corresponding HIV reservoir size (Darcis et al., 2017), indicating that the reservoirs size is one determinant of the cell capacity to produce virus but that this parameter alone is not sufficient. The patient-dependent heterogeneity in the responses to LRAs could be explained by patient characteristics such as genetic background, time to treatment initiation, duration and type of therapy and also by the gender-specificity, as recently proposed by the team of Jonathan Karn (Das et al., 2018). Sex-based differences in HIV-1 reservoir activity is characterized by a higher cell-associated HIV-1 RNA, higher plasma HIV-1 RNA, higher T-cell activation, and PD-1 expression in men compared to women (Scully et al., 2019). The group of Jonathan Karn has shown that the estrogen receptor-1 (ESR-1) is a key regulator of HIV-1 latency (Das et al., 2018). More specifically, antagonists of ESR-1 activate latent HIV-1 proviruses and potentiate HIV-1 reactivation by LRAs such as SAHA, TNFα, and IL-15, while ESR-1 agonists potently block HIV-1 reactivation. Despite a reduced inducible reservoir compared to men, women showed much higher levels of inhibition in response to TCR stimulation in the presence of ESR-1 agonists but exhibited a higher reactivation in response to ESR-1 antagonists when combined with SAHA than the group of male HIV^+^ individuals (Das et al., 2018). The circadian rhythm is an additional biological process that can affect HIV-1 transcription and reactivation (Chang et al., 2018). The circadian rhythm has been first suggested in the clinical trial testing short term administration of disulfiram (Elliott et al., 2015) as a parameter influencing HIV-1 transcription. The authors find an unexpected large variation in pre-dosing CA-US HIV-1 RNA which was statistically significantly higher immediately prior to the first dose of disulfiram than at the two previous time points without changes in HIV-1 DNA or plasma HIV-1 RNA (Elliott et al., 2015). Indeed, Sharon Lewin and colleagues have subsequently demonstrated a significant time-dependent variation in CA-US HIV-1 RNA in CD4^+^ T cells from HIV^+^ individuals on suppressive ART, a variation which is modulated by circadian regulator factors driving transcription from the viral LTR (Chang et al., 2018). Thus, in *ex vivo* studies, the time of blood collection could affect LRA reactivation potency and should be considered to improve latency reversal.

The multiplicity of mechanisms that regulate HIV-1 latency and the diversity of factors responsible for the heterogeneity of the latent HIV-1 reservoir most likely vary from one patient to the other and even from one cell to the other in a single patient. Indeed, several single-cell studies reported cell-to-cell variability of the latent reservoir (Baxter et al., 2016; Yucha et al., 2017) and the heterogeneity of cellular response to LRAs (Passaes et al., 2017). A recent single-cell transcriptome profiling study from Angela Ciuffi laboratory has demonstrated that latently-infected cells are transcriptionally heterogeneous and can be separated into two different cell clusters based on their cellular states (Golumbeanu et al., 2018). These distinct states correlated with the susceptibility to cellular activation and HIV-1 reactivation, highlighting that the cellular environment could also contribute to the success of HIV-1 reactivation strategies (Golumbeanu et al., 2018).

## Conclusion

In the frame of the “shock and kill” strategy, clinical trials using LRAs have so far produced unconvincing results. This strategy faces multiple barriers which prevent the complete eradication of replication competent viruses of the HIV-1 reservoir and must, therefore, be optimized. Targeting and reactivating latent cells is challenging due to the heterogeneous nature of the viral reservoirs. Recent studies demonstrating diverse responses of infected cells to LRAs point to their weak effect (Archin et al., 2012; Spivak et al., 2014; Elliott et al., 2015) and highlight the diversity of determinants responsible for the reservoirs’ heterogeneity that were demonstrated so far to be virus genetic background-(Norton et al., 2019), cell model-(Spina et al., 2013), cell type-(Baxter et al., 2016; Grau-Expósito et al., 2019; Kula et al., 2019), silencing mechanism-(Elliott et al., 2014; Yukl et al., 2018), tissue reservoir-(Elliott et al., 2014; Telwatte et al., 2018; Yukl et al., 2018), integration site-(Chen et al., 2017; Abner et al., 2018; Battivelli et al., 2018), patient-(Darcis et al., 2017; Yukl et al., 2018), and gender-(Das et al., 2018) specific. In addition, some studies demonstrate a heterogeneous effect of LRAs on NK cells (Garrido et al., 2016) and cytotoxic T-cell lymphocyte (Walker-Sperling et al., 2016) activity with conflicting observations, suggesting either an immunosuppressive effect or a reduced impact of LRA activity on cells sensing HIV-1 reactivation (Archin et al., 2012; Jones et al., 2014; Clutton et al., 2016; Walker-Sperling et al., 2016; Desimio et al., 2017, 2018). Moreover, prolonged ART treatment is associated with a significant reduction in the frequency of HIV-1-specific CD8^+^ T-cells (Gray et al., 1999; Casazza et al., 2001). Thus, as demonstrated by the group of Robert Siliciano, stimulating HIV-1-specific CTLs prior to reactivating latent HIV-1 should be considered for a successful eradication in future clinical trials (Shan et al., 2012). Another determinant of the effectiveness of a given LRA in reactivating and purging the viral reservoirs is its ability to efficiently induce latent HIV-1 by targeting not only transcriptional but also post-transcriptional mechanisms that need to be considered for a combined approach of more potent latency reversal. Additionally, an effective LRA needs to penetrate the multitude of HIV-1 tissue reservoirs and sanctuary sites. For instance, it has been shown that panobinostat did not sufficiently penetrate the central nervous system (Rasmussen et al., 2015) and romidepsin’s concentration in CSF of non-human primate was only 2% of the level found in plasma (Berg et al., 2004). Consequently, ensuring a better tissue penetration of LRAs by enhancing the drug delivery system, and most importantly strengthening the killing of the LRA-reactivated cells by stimulating CD8^+^ T responses are essential for the eradication strategy. Rational design of LRAs considering all these determinants is not possible at the moment, due to the lack of knowledge of all the cellular factors and pathways impacting HIV-1 gene expression and leading to productive viral replication. New approaches including single-cell technologies should be considered to understand why some cells respond to LRAs while others do not as this will be essential for improving the “shock and kill” strategy and hopefully reaching a cure. Moreover, due to the challenges that hinder the effectiveness of the “shock and kill” approach, some attention is also given to strategies aimed at completely suppressing HIV-1 transcription named the “block and lock.” In this context, the Tat inhibitor didehydro-cortistatin A (dCA) has shown its ability to inhibit *ex vivo* residual viral replication under ART and to prolong the time to viral reactivation after treatment interruption (Kessing et al., 2017), but some *in vitro* resistance mutations to dCA were reported (Mousseau et al., 2019). Currently, several drugs are identified for their ability to be used as LPA (Latency Promoting Agents) to ensure a functional cure (Suzuki et al., 2013; Wan and Chen, 2014; Vranckx et al., 2016; Hayashi et al., 2017; Jean et al., 2017; Debyser et al., 2018). Undeniably several strategies must be exploited in order to reach a functional cure. Dealing with the residual viremia and the contribution of ongoing viral replication in the reservoir’s replenishment is one of the major issues. If one of these strategies is promising its efficacy in clinic will be a long process and should, therefore, lead to the formation of a deep latency state preventing viral rebound after ART interruption.

## Author Contributions

AA-A, AK, GD, RV, and CV wrote the manuscript. SD, VG, PM, AM, and OR gave advice and suggestions for the writing of the manuscript.

## Conflict of Interest

The authors declare that the research was conducted in the absence of any commercial or financial relationships that could be construed as a potential conflict of interest.

## Abbreviations

5-AzaC: 5-azacytidine
5-azadC: 5-aza-2′deoxycytidine
5′LTR: 5′ Long Terminal Repeat
7SK snRNP: 7SK small nuclear ribonucleoprotein
APOBEC3A: Apolipoprotein B mRNA editing enzyme, catalytic polypeptide-like 3 A
ART: Antiretroviral therapy
ASP: Antisense protein
BETis: Bromodomain and Extraterminal (BET) bromodomain inhibitors
B-HIVE: Barcoded HIV ensembles
CA-US: Cell associated unspliced
CCR5/6/7: CC chemokine receptor 6/7
CGi: CpG island
CSF: Cerebrospinal fluid
CTIP2: COUP-TF (Chicken Ovalbumin Upstream Promoter Transcription Factor)-Interacting Protein 2
CXCR3/5: C-X-C Chemokine Receptor3/5
CNS: central nervous system
DCs: dendritic cells
DNA: Deoxyribonucleic acid
DNMT: DNA methyl-transferase
DSIF: DRB (5,6-dichloro-1-beta-D-ribofuranosylbenzimidazole) Sensitivity Inducing Factor
EDITS: Envelope Detection by Induced Transcription-based Sequencing
ESE_tat_: Exonic Splice Enhancer tat
ESR-1: Estrogen receptor-1
EZH2: Enhancer Of Zeste 2 Polycomb Repressive Complex 2 Subunit
fDCs: follicular dendritic cells
GALT: Gut-Associated Lymphoid Tissue
HDAC: histone deacetylase
HIV: human immunodeficiency virus
HLA-DR: Human Leukocyte Antigen – DR isotype
HMBA: Hexamethylene bisacetamide
HP1: heterochromatin Protein 1
HSCs: hematopoietic Stem Cells
IL-2/4/7/15: Interleukin 2/4/7/15
ITIM: Immunoreceptor Tyrosine-based Inhibitory Motif
KAP1: KRAB (Krüppel-Associated Box) domain-Associated Protein 1
LAG-3: Lymphocyte Activation Gene-3
LARA: latency and reversion assay
lncRNA: Long non-coding RNA
LRA: latency-reversing agents
MAPK: mitogen-activated protein kinase
MATR3: Matrin 3
mDCs: myeloid dendritic cells
miRNA: Micro ribonucleic acid
MMQO: 8-methoxy-6-methylquinolin-4-ol
Nef: negative regulatory factor
NELF: Negative Elongation Factor
NFAT: Nuclear Factor of Activated T-cells
NF-κB: Nuclear Factor-kB
PBMCs: peripheral blood mononuclear cells
PCAF: p300/CBP (CREB Binding Protein)-Associated Factor
PD-1: programmed cell death-1
PHA: phytohemagglutinin
PMA: phorbol myristate acetate
PML: promyelocytic leukemia protein
PRC2: polycomb repressive complex 2
PSF: polypyrimidine tract-binding protein associated splicing factor
PTB: Polypyrimidine Tract Binding protein
P-TEFb: Positive Transcription Elongation Factor b
qPCR: Quantitative polymerase chain reaction
QVOA: quantitative viral outgrowth assays
Rev: regulator of virion expression
RNA: ribonucleic acid
RNAPII: RNA polymerase II
RT-ddPCR: teverse transcription droplet digital PCR
SAHA: suberoylanilide hydroxamic acid
SIV: simian immunodeficiency virus
SMAC: second mitochondrial activator of caspases
SMYD2: SET (Suppressor of variegation, Enhancer of Zeste, Trithorax) and MYND (Myeloid-Nervy-DEAF1) domain-containing protein 2
Sp1: specificity protein 1
STAT5: signal transducer and activator of transcription 5
Suv39H1: Suppressor of variegation 3-9 homolog 1
TAR: transactivation response element
Tat: transactivator of transcription
T_CM_: central memory CD4^+^ T cells
TCR: T Cell Receptor
T_EM_: effector memory CD4^+^ T cells
T_fh_: T follicular helper cells
Th17: T helper cells 17
TIGIT: T-cell immunoreceptor with Ig ITIM domains
Tim-3: T cell immunoglobulin and mucin 3
TILDA: Tat/rev-induced limiting dilution assay
T_N_: Naïve CD4^+^ T cells
TNFα: tumor necrosis factor alpha
T_reg_: Regulatory CD4^+^ T cells
TRIM19: Tripartite Motif 19/22
TSA: trichostatin A
T_SCM_: stem cell-like memory CD4^+^ T cells
TSS: transcription start site
T_TD_: terminally differentiated CD4^+^ T cells
T_TM_: Transitional memory CD4^+^ T cells
UNAIDS: United Nations Programme on HIV and AIDS
UPF-1: up-frameshift protein 1
